# Biofilm: The invisible culprit in catheter-induced candidemia

**DOI:** 10.3934/microbiol.2023025

**Published:** 2023-05-11

**Authors:** Meiliyana Wijaya, Ryan Halleyantoro, Jane Florida Kalumpiu

**Affiliations:** 1 Department of Parasitology, School of Medicine and Health Sciences, Atma Jaya Catholic University of Indonesia, Jakarta, Indonesia; 2 Department of Parasitology, Faculty of Medicine, Universitas Diponegoro, Semarang, Indonesia; 3 Department of Parasitology, Faculty of Medicine, Pelita Harapan University, Banten, Indonesia

**Keywords:** candidemia, biofilm, catheter, extracellular matrix, treatment

## Abstract

Candidemia is the most common form of invasive fungal infection associated with several risk factors, and one of them is the use of medical devices, to which microbial biofilms can attach. Candidemia related to the use of peripheral intravascular and central venous catheters (CVC) is referred to as *Candida* catheter-related bloodstream infection, with more than 90% being related to CVC usage. The infection is associated with a higher morbidity and mortality rate than nosocomial bacterial infections. *Candida* spp. can protect themselves from the host immune system and antifungal drugs because of the biofilm structure, which is potentiated by the extracellular matrix (ECM). *Candida albicans* and *Candida parapsilosis* are the most pathogenic species often found to form biofilms associated with catheter usage. Biofilm formation of *C. albicans* includes four mechanisms: attachment, morphogenesis, maturation and dispersion. The biofilms formed between *C. albicans* and non-albicans spp. differ in ECM structure and composition and are associated with the persistence of colonization to infection for various catheter materials and antifungal resistance. Efforts to combat *Candida* spp. biofilm formation on catheters are still challenging because not all patients, especially those who are critically ill, can be recommended for catheter removal; also to be considered are the characteristics of the biofilm itself, which readily colonizes the permanent medical devices used. The limited choice and increasing systemic antifungal resistance also make treating it more difficult. Hence, alternative strategies have been developed to manage *Candida* biofilm. Current options for prevention or therapy in combination with systemic antifungal medications include lock therapy, catheter coating, natural peptide products and photodynamic inactivation.

## 1. Introduction

Biofilm is an attachment complex consisting of an assemblage of microorganism cells with a three-dimensional structure on a particular surface, and it occurs irreversibly. This attachment can occur on biotic (the host tissue) or abiotic surfaces (medical devices). The structural matrix can adhere to and be embedded in the surface due to microorganisms' extracellular polymeric substances (EPSs). One of the microorganisms that can produce biofilms to protect against the environment is *Candida* yeast, especially *Candida albicans* (*C. albicans*) [1],[2]. The formation of *Candida* biofilms is often a result of a source of nosocomial infections related to candidemia in intensively hospitalized patients because of its ability to grow to become attached to medical devices or host tissues [3],[4].

*Candida*, the most common cause of invasive fungal infection in hospitalized patients, has mortality rates that vary depending on the study population but are consistently high worldwide. In the case of neonates, the mortality rate reaches 35–60% [5], while, in pediatrics, it ranges between 10–35% [6], and 19.6–67% in adults [7],[8]. *Candida* is the primary agent of fungal sepsis, with a prevalence of 10–15%, and with 5% having severe sepsis or septic shock [9]. Among patients with septic shock, candidemia is the clinical condition with the highest mortality, ranging from 54 to 66% [10],[11]. The survival of patients with sepsis due to candidemia is strongly related to the early source control of medical devices used and appropriate antifungal treatment.

*Candida* bloodstream infection (CBSI) associated with catheter use has a high mortality rate, particularly in prolonged hospital stays in intensive care unit (ICU) patients [3],[12],[13]. The main culprit of CBSI related to catheter use is microbial biofilm formation [1],[14],[15]. Therefore, this review will focus on the discussion of the *Candida* biofilm formation mechanisms, especially in *C. albicans*, which is the primary source of *Candida* catheter-related bloodstream infection (CRCBSI), as well as the characteristics of biofilms by various non-*albicans Candida* (NAC) species, as well as mixed biofilms, and the principles of appropriate management and prevention.

## 2. Catheter-associated *Candida* biofilms

CBSI, also called candidemia, is the most common form of invasive fungal disease with *Candida* present in the blood [10],[12]. Several factors have been associated with the risk of candidemia in prolonged hospital stays in ICU patients, including using a catheter, total parenteral nutrition, systemic broad-spectrum antibiotic usage, *Candida* colonization history, history of surgery and being in immunosuppressive therapy. Moreover, underlying factors include immunocompromised persons with co-morbidities such as malignancy and hematological disorders, premature infants and low-birth-weight neonates; solid organ transplantation, neutropenia, extensive burns and renal dysfunction have also been reported as predispositions to CBSI [3],[12],[13],[16].

The epidemiology of candidemia varies according to geography, although it is dominated mainly by *C. albican*s, with the number of NAC infections increasing yearly [17]. Most invasive infections caused by NAC comprise *C. tropicalis* and *C. glabrata*, followed by *C. parapsilosis* and *C. krusei* [18]. *Candida albicans* and *C. parapsilosis* are the most frequent biofilm producers because of their ability of morphogenesis, facilitating the persistent infection associated with intravascular catheter use [19].

The CBSI related to peripheral intravascular and central venous catheters (CVC) is referred to as CRCBSI, with more than 90% being due to a CVC. The most frequent causative agents of CRCBSI are gram-positive bacteria, gram-negative bacteria and *Candida* yeasts [19],[20]. Catheters commonly used in ICU patients increase the risk of biofilm formation, which can lead to candidemia [15],[17],[19],[21]. Candidemia catheter-related infection has a high morbidity and mortality rate because it is associated with nosocomial infection, with higher mortality than bacterial biofilms, although it only accounts for about 5–10% of all sepsis cases and the incidence is rarely reported. In most cases, these lower prevalences caused by the classic diagnostic criteria used for sepsis and septic shock do not meet candidemia criteria [9],[11],[22]. Interestingly, Bassetti et al. [10] demonstrated that the use of a CVC is less commonly associated with intra-abdominal infection. These results support the observation of a recent murine model experiment that showed an increase in the number of *Candida* in the gut is associated with increased severity of bacterial sepsis due to the possibility of induction of a cytokine storm and/or decreased macrophage-killing activity [23].

## 3. *Candida albicans* biofilm formation mechanism

**Figure 1.**
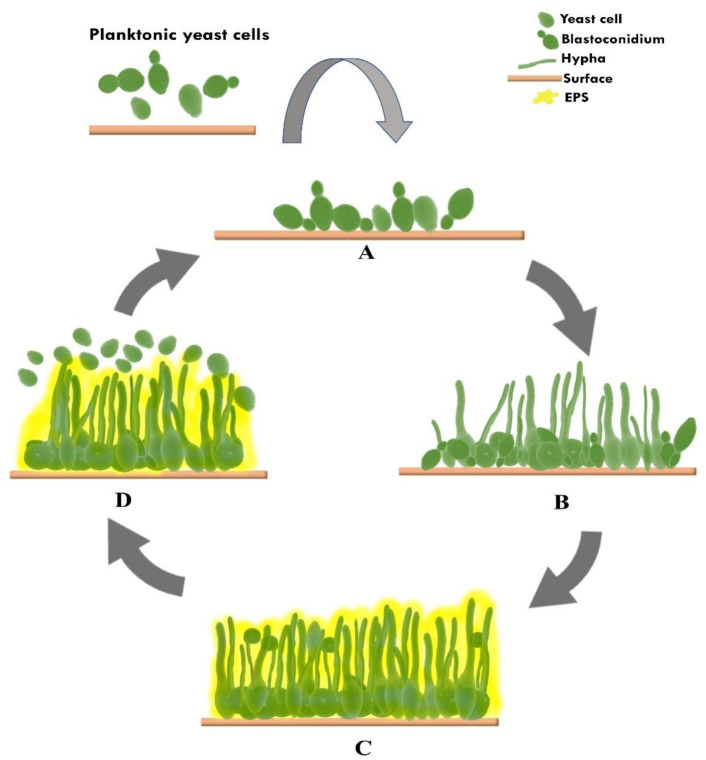
Schematic formation of *Candida albicans* biofilm. (A) Attachment to an abiotic or a biotic surface. (B) Proliferation and transition to hyphae. (C) Production of extracellular matrix and maturation of biofilm. (D) Detachment of yeast cells and dispersion.

*Candida albicans* is the most common commensal species isolated for candidemia, with high mortality rates being observed despite appropriate antifungal drug treatment [1],[3],[15],[24],[25]. The human fungal opportunistic pathogen *C. albicans* is a dimorphic fungus that can change morphologically from budding yeast to filamentous pseudohyphae and hyphae. This ability to undergo morphogenesis in the host helps it to adhere to or attack and form biofilms that contribute as virulence factors [26]–[28]. It also has a remarkable ability to form chlamydospores, which are thick-walled cells at the end of hyphae [29]. The formation of the *C. albicans* biofilm includes four main stages (Figure 1).

The first is the attachment process of *C. albicans* planktonic yeast cells to surfaces such as the epithelium, biomaterials or cellular aggregates [1],[25]. Furthermore, yeast cells will proliferate to form microcolonies and a basal layer of biofilm [25],[30]. The first phase in vitro takes approximately 11 hours. In comparison, in vivo experiments in a rat CVC have a shorter duration of 8 hours [1],[31]. The attachment process (Figure 1A) is mediated by fungal cell wall proteins called adhesins from three families, i.e., the agglutinin-like sequence (Als) family, the hyphal wall protein (Hwp) family and the individual protein file family F/hyphally regulated (Iff/Hyr) family [1],[4],[24],[32],[33]. Adhesins promote attachment to other cells, e.g., epithelial or microbial cells, or abiotic surfaces, by binding to specific amino acids or carbohydrate residues [1],[25]. The Als adhesin family of *C. albicans* consists of eight members, namely, Als1–7 and 9, whose proteins have a similar structure and contain N-terminal secretory signaling sequences. In *C. albicans*, Als1, Als3 and Als5 were most involved in the attachment process to surface and host cells [25],[32]–[34]. The Hwp adhesin families are required for biofilm development, including Hwp1 and Hwp2, as well as for enhanced adherence to polystyrene 1 (Eap1), which is repressed by Tup1 (Rbt1), and yeast wall protein (Ywp1) (Table 1). Hwp 1 is a major hyphal cell wall protein and the most studied cell wall mannoprotein in *C. albicans* germ tube form and hyphae, which promote attachment to the host surface [25],[32],[33]. In addition, once attachment occurs, persister cells that are phenotypic variants of wild-type cells and constitute only a tiny proportion of the biofilm population will also form, although their proportion will remain constant during biofilm development. These cells are highly associated with cases of remarkable tolerance because they do not grow but can survive high doses of antifungal treatment [35].

The second stage is the increase in the number of yeast cells (proliferation), and then the transitional stage or morphological modification, where yeast cells change into hyphae (Figure 1B) [1],[25]. Biofilm development in this stage involves the transcriptional regulators: Bcr1, Brg1, Efg1, Tec1, Ndt80 and Rob1 (Table 1) [24],[32],[33]. The attachment of hyphae is specifically mediated by Hwp1, a gene that is required for hyphal development, mating and the maintenance of biofilm integrity [36]. The hypha form has an essential contribution to the overall architectural stability of the biofilm and acts as a supporting scaffold for fellow yeast cells, pseudohyphae, hyphae and other microbial cells in the context of polymicrobial biofilms. Thus, this morphogenesis in *C. albicans* and the ability of these hyphae to adhere to one another is very important for developing and maintaining biofilms [28],[33],[36].

The third stage is the maturation process, in which fungal cells called sessile cells become organized and trapped in the EPS, where they produce to form the three-dimensional architectural matrix of the mature biofilm structure (Figure 1C) [37]. The EPS has a hydrated, gel-like three-dimensional structure, immobilizing some biofilm cells to support structural integrity [1],[37]. The extracellular matrix (ECM) formed is an organic polymer consisting of proteins (55%), carbohydrates (25%), lipids (15%) and extracellular deoxyribonucleic acid (eDNA) (5%) [15],[24],[25],[37]. There are more than 500 proteins identified in the biofilm matrix, with the majority of glycolysis enzymes that may degrade extracellular biopolymers acting as a source of energy and heat shock protein (Hsp) [24],[37]. The dominant polysaccharide in ECM biofilms of *C. albicans* is the mannans glucan complex, namely, ∝-1,2 branched ∝-1,6 mannan, which is associated with linear β-1,6 glucan and a small amount of β-1,3 glucan. Although β-1,3 glucan is only a small component, it plays a significant role in biofilm resistance to antifungals, especially azole groups, by inhibiting drug diffusion. The profile lipid of the ECM is composed of glycerolipids (99.5%), for much fewer sphingolipids (0.5%). In addition, the only sterol detected was ergosterol, though at low concentrations [37]. Another major contributor to the stability and growth of *C. albicans* biofilms in the ECM is eDNA with predominantly random non-coding sequences. The proposed mechanisms for releasing eDNA into the matrix include cell lysis, quorum sensing (QS) and excretion from DNA-containing vesicles [32],[37]. The QS also mediates communication between sessile cells in the morphogenesis and biofilm formation of *C. albicans*. The molecule of QS that plays a role is tyrosol, which induces filamentation and biofilm formation, while farnesol plays the opposing role [38].

The ECM is partially self-produced and secreted by *C. albicans* cells within the biofilm but may also contain aggregates from the surrounding environment. These aggregates include *C. albicans* cells and lysed host cells, as well as host-specific cells recruited to the region and fused into biofilms, such as epithelial cells and neutrophils. Therefore, the composition of the ECM may vary depending on the location of the biofilm in the host [2]. The ECM's unique structure can protect innate host immune defenses, especially against neutrophils and monocytes [25],[37]. Monocytes can phagocytize *C. albicans* planktonic cells, but not sessile cells in biofilms. Furthermore, neutrophils cannot eliminate mature biofilms due to a failure to release extracellular traps, as well as failed activation due to the inhibition of matrix glucans, which act as decoy mechanisms and cannot activate reactive oxygen species [2],[4],[37]. Two transcription factors involved in regulating ECM production for *C. albicans* biofilms is Zap1 and Rlm1 (Table 1) [32],[33].

The ECM in biofilms also plays an essential role in antifungal resistance, although it is multifactorial and based on different mechanisms. One of the mechanism is demonstrated by the dominant role of component β-1,3 glucan in biofilm resistance to fluconazole and amphotericin B due to sequestration of antifungal matrix components that prevent the drug from reaching cellular targets [37],[39]–[41]. Another matrix component that plays a role is eDNA; Martins et al. [42] reported that the addition of DNase increased the susceptibility of some antifungals, such as echinocandins and polyenes, but not fluconazole.

The fourth stage is the dispersal of mature biofilms (Figure 1D) by releasing budding daughter yeast cells as the elongated cells that are not attached to colonize other parts to form a secondary infection site [1],[15],[30],[38]. Detachment of yeast-like cell buds from the hyphae's upper biofilm layer allows *C. albicans* to disseminate to the bloodstream and cause invasive disease. Although the released cells resemble yeast, they have a unique transcriptional profile and differ from planktonic cells or biofilms by showing increased virulence and causing more damage to endothelial cells [15],[30]. These cells are believed to play a significant role in the pathogenesis of vascular catheters and other infections that cause disseminated disease [15]. The genes that regulate the process of dispersal of yeast cells are Ume6, Pes1 and Nrg1 (Table 1) [32],[33].

**Table 1.** Factors contributing to the formation of *Candida albicans* biofilm [1],[4],[24],[32]–[34].

Gene name	Description	Stage
Als	Consists of eight large cell surface glycoproteins: Als1–7,9; with Als 1, Als3 and Als5 as the most involved in the attachment process	Attachment
Hwp	Hwp1, Hwp2 and Rbt1 (repressed by Tup1) belong to this gene family, which is required for adhesion to host cell surface proteins and cell aggregationHwp1 is also required for hyphal formation	Attachment and transitional
Bcr1	Important in the adhesion process of yeast cells to the surface, and also acts as a trancription factor for upregulation in Hwp1 expression	Attachment and transitional
Efg1		
Brg1	Transcription factor to promote hyphal formation	Transitional
Rob1		
Tec1		
Ndt80		
Zap1	Negative regulator of ECM production	Maturation
Rlm1	Positive regulator of ECM production	
Nrg1	Overexpression leads to increased dispersion cells	Dispersion
Pse1		
Ume6	Overexpression leads to reduced dispersion cells	Dispersion

## 4. Characteristics of various non-*albicans Candida* species forming biofilms

The formation of biofilms in *Candida* spp. is different in the morphology, characteristics of the ECM and their ability of antifungal resistance [29],[43]. The most pathogenic species often found to form biofilms is *C. albicans*, which will be discussed in the formation mechanism. Furthermore, NAC and some species formerly known as genus *Candida*, are important in cases of candidemia and have the ability to form biofilms, including *C. tropicalis*, *C. parapsilosis*, *C. dubliniensis*, *C. auris*, *Nakaseomyces glabrata* (also called *C. glabrata*), *Pichia kudriavzevii* (also called *C. krusei)* and *Meyerozyma guilliermondii* (also called *C. guilliermondii*) [29],[43]–[45]. Nonetheless, the exact mechanism of these NACs and their morphogenesis on pathogenicity in biofilm formation has not been fully characterized. The formation of biofilms in *Candida* spp. is different in terms of morphology, characteristics of the ECM and their ability of antifungal resistance [29],[43]. The differences in these biofilms' structures and the ECMs' composition are affected by the environmental conditions experienced by *Candida* spp. One crucial factor is the substrate of the abiotic surface to which the biofilm is attached. Several substrates are used to study *Candida* biofilms in vivo and in vitro, such as polystyrene, polyurethane, polymethylmethacrylate, polyvinyl chloride (PVC), cellulose, silicone and latex elastomer [1].

*Candida tropicalis* has emerged as the second or third most common and virulent agent of candidemia, and it is particularly relevant in catheter-associated urinary tract infections with high biofilm-forming capacity [1],[3],[44]. The biofilm structure is composed of a dense network of yeast cells, pseudohyphae and true hyphae enclosed in an ECM [33],[43]. *Candida tropicalis* is also a robust and efficient biofilm producer of the EPS's high thickness [44]. The ECM composition consists of low carbohydrate and protein levels, hexosamine, phosphorus and uronic acid [1],[33],[44]. The gene that plays a role in the adhesion process is ALS1–16, while the maturation process is carried out with Efg1 [33]. *Candida tropicalis* can produce biofilms significantly on silicon, PVC and polyurethane catheters, and it is reported to be resistant to azoles, especially to fluconazole [44],[46],[47].

The increasing use of medical devices and parenteral nutrition has made *C. parapsilosis* the most frequently isolated NAC fungal organism associated with catheter use, especially in critically ill neonates and transplant recipients [48]–[51]. The hypothesis of a high rate of *C. parapsilosis* infection in neonates is thought to be associated with its ability to utilize fat and fatty acids as the primary energy source in critically ill patients; it is believed to be capable of growing in a solution with high glucose concentration, especially in neonates who often receive parenteral nutrition [49],[50],[52]. This fungus is also the causative agent of several catheter-related infections due to being frequently found on the skin of healthy humans [48],[53]. Their characteristics and ability to switch into pseudohyphae and form biofilms on prosthetic materials, such as CVCs, could create nosocomial outbreaks with high mortality. *Candida parapsilosis* biofilm consists of clumping blastoconidia, yeast cells and pseudohyphae, with a thin ECM consisting of high carbohydrate levels with low protein. The formation of a *C. parapsilosis* biofilm is best demonstrated on a PVC catheter. Several genes involved in forming the *C. parapsilosis* biofilm were also found in the *C. albicans*, such as Bcr1, Efg1 and Hwp1 [1],[33]. Moreover, *C. parapsilosis* isolates have been found to increase resistance to azoles, especially fluconazole, at a rate five times higher than in *C. albicans*; they also have intrinsic resistance to echinocandins [54].

*Candida dubliniensis* is a species with many similar phenotypic characteristics to *C. albicans*, and it was often misidentified due to its ability to form germ tubes, chlamydospores and hyphae, but with fewer virulence genes than *C. albicans* [55],[56]. These two types of *Candida* are also known to secrete farnesol, a QS molecule that inhibits *Candida* biofilm development through the inhibition of filamentation, which in turn may decrease its potential to invade deeper tissues [1],[57]. Nonetheless, genes involved in biofilm formation have not yet been explored. In addition, *C. dubliniensis* is much less common in the microbiome, which correlates with the low prevalence of *C. dubliniensis* as invasive candidiasis [55],[58]. The differences between the two species include the following: *C. dubliniensis* can grow well at 30 °C and 37 °C, like *C. albicans* in Sabouraud dextrose agar culture media; however, it does not grow at all at 42 °C [55]. The prevalence of isolates of this species is very high in the oral cavity of patients infected with human immunodeficiency virus (HIV) or acquired immune deficiency syndrome (AIDS), probably due to its increased ability to adhere to buccal epithelial cells. However, the underlying reason is unclear [55],[56],[58]–[60]. *Candida dubliniensis* has also shown resistance to fluconazole, due to its increased use as prophylaxis in HIV/AIDS patients [54],[55],[60].

*Candida auris* survives well on fomites, medical equipment and surfaces in the hospital environment with chlorhexidine clearance [61]–[63]. A characteristic of *C. auris* in biofilms is that it is also less sensitive to several disinfectants, such as hydrogen peroxide, povidone-iodine and sodium hypochlorite [45]. This fungus can also spread between patients, mainly in hospitalized patients with severe illness, or with the prolonged use of indwelling devices, thus later causing candidemia with high mortality. The *C. auris* biofilms consist primarily of budding yeast cells, which are round/oval with a small ECM; and, pseudohyphae are rarely found [64]. The ECM component of *C. auris* contains many mannan-glucan polysaccharides [45],[62]. Along with its discovery and characteristics, it has become an epidemic in several healthcare centers globally, which is very important because it has multidrug-resistant properties to antifungals [61],[65]. Resistance has been demonstrated, primarily to fluconazole, with variable susceptibility to other azoles, amphotericin B and echinocandins [45],[54],[63].

*Nakaseomyces glabrata* (*C. glabrata*) is some regions' second most common cause of invasive disseminated candidiasis; it is a poor biofilm producer in a catheter, but is strong in oral isolates [18],[66],[67]. It also showed to form biofilms on different medical devices [68]–[70]. These fungi are not dimorphic due to the inability of this species to form filaments of either true hyphae or pseudohyphae as commensals or pathogens; thus, the formed biofilm consists of only monolayer or multilayer blastoconidia that are tightly packed and wrapped in a thin ECM [1],[29],[33]. Despite that, *C. glabrata* has an ECM matrix component with high carbohydrate and protein content. The adherence mechanism is mediated by epithelial adhesins that have a similar structure to the Als proteins [70]. Estivill et al. [47] showed that *C. glabrata* biofilm formation was best on PVC. Furthermore, many *C. glabrata* strains that cause septicemia are resistant to antifungal azoles, particularly fluconazole, and strains with mutations in the FKS gene show resistance to echinocandins [1],[54].

*Pichia kudriavzevii* (*C. krusei*) is often found in patients with underlying hematological malignancy [18]. This fungus can produce intermediate biofilms on PVC and polyethylene surfaces [29],[71]. Unfortunately, the significance of *C. krusei* biofilms and their morphogenesis for pathogenicity has not been fully characterized. Like *C. albicans*, *C. krusei* has the property of thermomorphism, that is, it can change the shapes of blastoconidia and pseudohyphae into hyphae at 37 °C so that the primary regulatory network that regulates dimorphism related to biofilm formation is also similar [71]. *Candida krusei* is intrinsically resistant to fluconazole, and infections due to this species commonly occur in patients receiving fluconazole prophylaxis and who have a prior history of neutropenia [18],[71].

## 5. Characteristics of mixed-species biofilm

Biofilm infection can be caused by a single microbial species or a mixture of different species, but more studies have focused on a single species in various host niches [25],[72],[73]. Polymicrobial interactions can alter the expression of virulence factors; thus, the biofilms that form are often more resistant to antimicrobial drugs [72]. In addition, polymicrobial interactions can also affect the host's immune response and the outcome of infection because the immune response to one species can affect immunity to other organisms [72],[73]. The interaction between *Candida* spp., especially *C. albicans* with microbes found on biotic and abiotic surfaces, which are very varied and complex, consists of synergistic or antagonistic relationships [25],[72]–[74]. The interaction of *C. albicans* with other microorganisms can occur through coaggregation and coadhesion processes [74].

*Candida* polymicrobial biofilms can associate synergistically or antagonistically with gram-positive and gram-negative bacteria. Gram-positive bacteria such as *Staphylococcus aureus* and *Streptococcus mutans*, which are found in the oral mucosa of denture users, as well as gram-negative bacteria such as *Escherichia coli* in the digestive tract and bladder mucosa, showed a synergistic relationship, while the antagonistic relationship is shown in gram-positive bacteria such as *Lactobacillus* spp. in the vulvovaginal environment and *Pseudomonas aeruginosa*, which are frequently co-isolated from contaminated catheters and chronic lung infections [25],[72],[73],[75].

The formation of mixed species biofilms involving *C. albicans* and bacteria has been described extensively; however, little has addressed intra-genus interactions between *Candida* species. *Candida albicans* has been studied more extensively than any other NAC species, although the role of NAC species has received increasing attention over time by clinicians [25]. Unfortunately, the interactions, except for *C. albicans* on host responses, are poorly characterized. Abrantes et al. [76] reported that *Candida* mixed biofilm interactions using an impedance-based biofilm monitoring system showed that the maximum cell adherence index increased over time in most mixed biofilms more than in monocultures, except *C. albicans*. Cell enhancement can be due to synergistic activity between NAC species that restore matrix structure and function, which allows species deficient in certain carbohydrates to be supplemented by their neighbors with different carbohydrates. When using the conventional crystal violet staining, the most biofilm formation was observed in mixed *C. albicans* and *C. tropicalis* biofilms, with the lowest being observed for the *C. glabrata/ C. parapsilosis* combination [76].

*Candida albicans* forms antagonistic interactions with NAC species in mixed biofilms, while the combination will benefit NAC over *C. albicans* since this species produces more significant amounts of the ECM when in a monoculture [77]–[79]. A study by Pathirana et al. [80] showed that, out of five NAC species (*C. tropicalis*, *C. dubliniensis*, *C. parapsilosis*, *C. lusitaniae* and *C. krusei*), only *C. tropicalis* and *C. dubliniensis* were able to attach to *C. albicans* hyphae. These two NAC species share a close phylogenetic relationship with *C. albicans* and the ability to form true hyphae. This mixed biofilm shows a growth advantage for both NAC species and not for *C. albicans*. Furthermore, Santos et al. [77] showed that, among the NAC species, *C. krusei* had the highest inhibitory activity against the in vitro biofilm model of *C. albicans*. These results suggest that there may be nutritional competition between these species, or *C. krusei* generating signaling molecules that inhibit *C. albicans* growth. Another study from Barros et al. [81] regarding the combination biofilms of *C. albicans* with *C. krusei* indicates a competitive antagonistic interaction between these two species by altering or inhibiting the mechanisms involved in the in vitro adherence and formation of *C. albicans* biofilms. *Candida krusei* can inhibit the expression of the Als1, Als3, Hwp1, Bcr1, Efg1 and Tec1 genes of *C. albicans* during in vitro biofilm production. Meanwhile, biofilms mixed with *C. albicans* and *C. glabrata* also showed a decrease in *C. albicans* biofilm mass; however, there was a slight increase in the expression of Als3, Hwp1, Tec1 and Bcr1 from *C. albicans*; thus, this study suggests a neutral relationship, or even synergism [81].

## 6. Update on strategies to combat *Candida* biofilm infections

Efforts to control the formation of *Candida* spp. biofilms are still challenging. Biofilms can readily colonize on permanent medical devices used by patients, such as intravascular catheters, urinary catheters or prosthetic heart valves, where prolonged use, especially in critically ill patients, increases the risk of developing candidemia, resulting in sepsis [1],[9],[15]. In patients with CRBSI caused by *Candida* spp., catheter removal is recommended for decreased mortality [82]–[84]. However, occasionally, catheter removal is impossible in patients such as neonates with extremely low birth weight, patients with surgical catheter implants without other available vascular access or those with severe coagulopathy [83],[85]. The availability of antifungal drug options for treating systemic candidiasis is limited to azoles, echinocandins and polyenes [86]. Moreover, each of these groups has its challenges; where the azole group acts as a fungistatic rather than a fungicidal drug for *Candida*, there is increased resistance due to general and long-term use, and several *Candida* species show intrinsic resistance to fluconazole [54],[87]. Echinocandins appear to be the answer for azole-resistant yeasts, but prolonged exposure of azole drugs to *Candida* isolates has reduced its susceptibility, especially in immunocompromised patients with recurrent candidemia [54]. Amphotericin B is considered the ultimate therapy, although it has infusion-related toxicity as a side effect, especially in conventional formulations [88]. Hence, numerous prevention and therapeutic methods have evolved to replace or combine with systemic antifungal treatment.

Prevention of biofilm formation related to catheter use currently has two methods, i.e., lock therapy and catheter coating [1],[14]. The lock therapy methods inhibit *Candida*'s attachment to the surface, which is the initial stage of biofilm formation. In this approach, prior to contact with the patient, it is possible to inject high concentrations of antimicrobial agents (usually from 100 to 1000 times the minimal inhibitory concentration (MIC)) into the lumen of the catheter and leave them for several hours or days [83],[89]. The advantage of this method is that it prevents systemic toxicity because it only acts in the catheter; yet, this strategy against *Candida* can fail to eradicate it if biofilms are formed on the extraluminal of the catheter [1],[14].

Based on in vitro and in vivo studies, the most promising antifungal in lock therapy against *Candida* biofilms are echinocandins and amphotericin B; however, with the azole group, they are less effective [89]–[91]. Echinocandins inhibit the synthesis of β-(1,3)-d-glucan, an essential component of fungal cell walls, with anti-biofilm activity that is effective even at low concentrations approaching the MIC [54]. Caspofungin, micafungin and anidulafungin are echinocandins used in lock therapy [83]. Caspofungin is the class of echinocandin most widely explored as a type of lock therapy owing to its anti-biofilm activity against *C. albicans*, *C. auris*, *C. lusitaniae*, *C. glabrata* and *C. guilliermondii* [83],[92]. Interestingly, micafungin can reduce the metabolic activity of the *C. albicans* biofilm by up to 98% if combined with 20% ethanol and 800 µg/mL doxycycline. In addition, micafungin can effectively eliminate catheter-associated candidemia in male infants if combined with 0.3 mL of 70% ethanol [93].

Furthermore, micafungin and anidulafungin are recommended for *C. glabrata* since they show much higher activity as compared to liposomal amphotericin B (LAmB)-based in vitro studies [94]. Amphotericin B acts by binding to ergosterol in the fungal cell membrane, which induces the formation of aqueous pores and the sequestration of ergosterol, which triggers oxidative damage. [54],[91]. An in vivo study by Fujimoto and Takemoto [91] showed that using LAmB was more effective in eradicating *Candida* biofilms in catheters when given a combination of systemic and lock therapies, as compared to micafungin. The LAmB administration from in vitro studies also shows superior activity for *C. albicans* and *C.tropicalis*, while, for *C. parapsilosis*, both micafungin and LAmB are comparable [91].

Methods of coating on the surface of a catheter with biomaterial innovations, such as antifungals, polymers and nanoparticles, have been explored [1],[95]. Caspofungin is the antifungal class of echinocandins, with primary amines often used as coating materials to prevent fungal cell attachment using covalent interactions [96]–[99]. Interestingly, Naderi et al. [100] demonstrated that coating with other echinocandins, such as anidulafungin and micafungin, can also eliminate 10^6^ CFU/cm^2^ inoculation of *C. albicans* and prevent biofilm formation and conversion to hyphae. Although surface coating with Food and Drug Administration (FDA) approved antifungal is promising, there is concern about increasing resistance in this way. An alternative was developed by using small molecules and peptides with antifungal and anti-biofilm properties [95]. Filastatin is a small-molecule inhibitor that effectively inhibits the attachment of *C. albicans* to the surface of medical devices [101]. Biomedical surfaces coated layer-by-layer and combined with the antifungal 14-helical β-peptide structure can efficiently eliminate and inhibit biofilm formation when administered in bulk solution to planktonic populations of *C. albicans*, or when released slowly from polymer coatings containing embedded β-peptides [102],[103]. Polymer-based catheter coatings also possess antifungal and/or antibiofilm properties, with the advantage of being less susceptible to resistance than small-molecule antifungals, making them an effective alternative in candidemia treatment. Chitosan is a bioactive polymer from naturally derived polysaccharides; it is widely incorporated into hydrogels and used as a coating to prevent *Candida* attachment and the formation of biofilms [104]. Another type of coating material that has developed rapidly in recent years is chitosan-coated polymeric silver and gold nanoparticles, which have anti-biofilm effects and may increase the bioavailability of antifungal drugs. In addition, they have the advantage of being able to penetrate EPSs and target fungal cells so that they can be used as *Candida* biofilm therapy [105],[106].

Another alternative developed to treat *Candida* biofilms is natural peptide products from plant extracts, microbes, insects or other sources [107],[108]. Antifungal peptides consist of various classes, with the most widely studied classes related to fungal biofilms being defensins, cathelicidins and histatins. These compounds act by preventing the formation of biofilms and eradicating preformed ones through various mechanisms, including disrupting fungal membrane integrity, inhibiting the adhesion of planktonic fungal cells to the surface, disrupting gene regulation and inducing the production of reactive oxygen species [102]. While this alternative is promising, the toxicity of these compounds has not been established, and the doses tolerated in patients need to be studied further [109].

Recently, an alternative approach using photodynamic inactivation (PDI) to eliminate *Candida* biofilms has been developed with a different class of non-toxic dyes called photosensitizers (PSs) [1],[110],[111]. This technique uses visible light at the appropriate wavelength and a PS to induce cell damage and death through apoptosis or necrosis in target cells. When the PS is activated by light, it will react to oxygen and make the microorganisms produce reactive oxygen species, causing damage to the DNA and increased cell membrane permeability [111]–[113]. Various classes of PSs have been reported for PDI in *C. albicans*, including curcumin, methylene blue (the most commonly used), porphyrin and toluidine blue [110],[111]. This approach is effective against drug-resistant strains and does not show the potential to develop drug resistance [110],[114]. In addition, it has been proven that combining PDI with antifungal agents can increase the planktonic killing power of *C. albicans* [110],[115],[116]. All of the strategies for the prevention and therapy of catheter-associated *Candida* biofilms that are suggested to replace or be combined with systemic antifungal treatment are summarized in Table 2.

**Table 2.** Strategies to combat catheter-associated *Candida* biofilm infection.

Methods	Recommendation and notes	References
Lock therapy	Echinocandins (caspofungin, micafungin and anidulafungin) and amphotericin B were used as lock solutions (intraluminal) for both prevention and treatment of catheter-related infections.	[85]–[87]
Catheter coating	Caspofungin is the most commonly used in direct surface coating for prevention. Small molecule (filastatin), polymers (chitosan) and nanoparticles (silver or gold) demonstrated antibiofilm activity for alternative coating.	[92]–[95],[97],[100]–[102]
Natural peptide products	Defensins, cathelicidins and histatin are the alternative peptides from plant extracts, microbes, insects or other sources that show antifungal activity to inhibit *Candida* biofilm.	[98]
Photodynamic inactivation	Methylene blue is the most commonly used PS to inhibit the viability of the biofilm produced by *Candida*.	[106],[107]

## 7. Conclusions

*Candida* is a common source of nosocomial infection due to its ability to form biofilms on medical devices, especially CVCs in ICU patients. *Candida albicans* and *C. parapsilosis* are species that are often isolated in association with biofilms due to their ability in morphogenesis to facilitate the occurrence of persistent infections associated with catheter use. This review also highlights the complexity of *Candida* species biofilm formation and the different characteristics between *C. albicans* and NAC species, such as the morphology, unique ECM composition and antifungal resistance. Therefore, recent management and prevention of *Candida* biofilm, including lock therapy, catheter coating methods, natural peptides and PDI, can be effective despite the limited antifungal armamentarium and increasing drug resistance.
